# Reviewer acknowledgement 2014

**DOI:** 10.1186/s12977-015-0143-y

**Published:** 2015-02-15

**Authors:** Andrew Lever, Mark Wainberg

**Affiliations:** University of Cambridge, Cambridge, UK; McGill University AIDS Centre and Jewish General Hospital, Montreal, Canada

## Abstract

*Retrovirology* would like to sincerely thank the following for giving their time and expertise to review manuscripts for the journal in 2014. Their support for the journal is greatly appreciated.

**Ana Abecasis**

Portugal

**Philippe Afonso**

France

**Daniel Aguiar**

Brazil

**Christopher Aiken**

United States of America

**Jan Albert**

Sweden

**Jose Alcami**

Spain

**Ghalib Alkhatib**

United States of America

**Neil Almond**

United Kingdom

**Zandrea Ambrose**

United States of America

**Emma Anderson**

United Kingdom

**Valgerdur Andresdottir**

India

**Adam Arkin**

United States of America

**John Armour**

United Kingdom

**Elmostafa Bahraoui**

France

**Norbert Bannert**

Germany

**Francis Barin**

France

**Eric Barklis**

United States of America

**Stéphane Basmaciogullari**

France

**Andreas Baur**

Germany

**Rupert Beale**

United Kingdom

**Bruno Beaumelle**

France

**Robert Belshaw**

United Kingdom

**Serge Benichou**

France

**Yamina Bennasser**

France

**Steven Bensinger**

United States of America

**Clarisse Berlioz-Torrent**

France

**Neil Berry**

United Kingdom

**Lionel Berthoux**

Canada

**Dorothee Bienzle**

Canada

**Fabien Blanchet**

France

**Julia Blanco**

Spain

**Jonas Blomberg**

Sweden

**Willy Bogers**

Netherlands

**Morgane Bomsel**

France

**Kathleen Boris-Lawrie**

United States of America

**Pedro Borrego**

Portugal

**Fadila Bouamr**

United States of America

**Paul Boyer**

United States of America

**Christian Brander**

Spain

**Abraham Brass**

United States of America

**Jason Brenchley**

United States of America

**Bluma Brenner**

Canada

**Mark Brockman**

Canada

**Kristina Broliden**

Sweden

**James Bruce**

United States of America

**Michael Bukrinsky**

United States of America

**Dennis Burton**

United States of America

**Florence Buseyne**

France

**Paul U Cameron**

Australia

**Edward Campbell**

United States of America

**Carol Carter**

United States of America

**Nicoletta Casartelli**

France

**Francesca Ceccherini-Silberstein**

Italy

**Shan Cen**

China

**Rob Center**

Australia

**Theresa L Chang**

United States of America

**Nathalie Chazal**

France

**Linda Chelico**

Canada

**Bing Chen**

United States of America

**Benjamin Chen**

United States of America

**Irvin Chen**

United States of America

**Cecilia Cheng-Mayer**

United States of America

**Peter Cherepanov**

United Kingdom

**Francesca Chiodi**

Sweden

**Nicolas Chomont**

United States of America

**Frauke Christ**

Belgium

**Tomas Cihlar**

United States of America

**Andrea Cimarelli**

France

**Angela Ciuffi**

Switzerland

**Paul Clapham**

United States of America

**Francois Clavel**

France

**Mario Clerici**

Italy

**Alan Cochrane**

Canada

**John Coffin**

United States of America

**Eric Cohen**

Canada

**Ronald Collman**

United States of America

**Rafael Contreras-Galindo**

United States of America

**Pierre Corbeau**

France

**Barbara Coulson**

Australia

**Robert Craigie**

United States of America

**Alexandra Cribier**

France

**Suzanne Crowe**

Australia

**Nathan Cummins**

United States of America

**Anthony Cunningham**

Australia

**Atze Das**

Netherlands

**Siddhartha Datta**

United States of America

**Miles Davenport**

Australia

**Rob de Boer**

Netherlands

**Hugues de Rocquigny**

France

**Thushan de Silva**

United Kingdom

**Gregg Dean**

United States of America

**Zeger Debyser**

Belgium

**Nathalie Dejucq-Rainsford**

France

**Gregory Del Prete**

United States of America

**Joachim Denner**

Germany

**Paul Denton**

Denmark

**Cynthia Derdeyn**

United States of America

**Diane Descamps**

France

**Benjamin Descours**

France

**Jeffrey DeStefano**

United States of America

**Felipe Diaz-Griffero**

United States of America

**Katie Doores**

United Kingdom

**Victoria D'Souza**

United States of America

**Diako Ebrahimi**

Australia

**Dirk Eggink**

Netherlands

**Maryam Ehteshami**

United States of America

**Michael Emerman**

United States of America

**Stephane Emiliani**

France

**Alan Engelman**

United States of America

**Le Rouzic Erwann**

France

**Jose Este**

Spain

**Oliver Fackler**

Germany

**Hung Fan**

United States of America

**Nuno Faria**

United Kingdom

**Ariberto Fassati**

United Kingdom

**Maurizio Federico**

Italy

**Jacques Fellay**

Switzerland

**Andrés Finzi**

Canada

**Donald Forthal**

United States of America

**Philippe Fossé**

France

**Alan Frankel**

United States of America

**Eric Freed**

United States of America

**Simon Frost**

United Kingdom

**Jun-ichi Fujisawa**

Japan

**Axel Fun**

United Kingdom

**Susanne Gabrielsson**

Sweden

**Yonatan Ganor**

France

**Michael Gantier**

Australia

**Feng Gao**

United States of America

**Victor J Garcia**

United States of America

**Gerardo Garcia-Lerma**

United States of America

**Anne Gatignol**

Canada

**Howard Gendelman**

United States of America

**Anna Maria Geretti**

United Kingdom

**Matthias Geyer**

Germany

**Mauro Giacca**

Italy

**Robert Gifford**

United Kingdom

**Paul Goepfert**

United States of America

**Stephen Goff**

United States of America

**Maureen Goodenow**

United States of America

**John Goodier**

United States of America

**Nilu Goonetilleke**

United States of America

**Miroslaw Gorny**

United States of America

**Paul Gorry**

Australia

**Heinrich Gottlinger**

United States of America

**Duane Grandgenett**

United States of America

**Lachlan Gray**

Australia

**Patrick Green**

United States of America

**Harriet Groom**

United Kingdom

**John Guatelli**

United States of America

**Hillel Haim**

United States of America

**Lou Hammarskjold**

United States of America

**David Harrich**

Australia

**Richard Harrigan**

Canada

**Reuben Harris**

United States of America

**Alex Harwig**

Netherlands

**Theodora Hatziioannou**

United States of America

**Johnny He**

United States of America

**Sonya Heath**

United States of America

**Jonathan Heeney**

United Kingdom

**Jiri Hejnar**

Czech Republic

**Georges Herbein**

France

**Ann Hessell**

United States of America

**Vanessa Hirsch**

United States of America

**Carol Hlela**

South Africa

**J. Robert Hogg**

United States of America

**Margaret Hosie**

United Kingdom

**Agnes Hotz-Wagenblatt**

Germany

**Selwyn Joseph Hurwitz**

United States of America

**Hendrik Huthoff**

United Kingdom

**Jun-ichiro Inoue**

Japan

**Fumitoshi Ishino**

Japan

**Astrid Iversen**

United Kingdom

**Yasumasa Iwatani**

Japan

**Nuria Izquierdo-Useros**

Spain

**Pooja Jain**

United States of America

**Martin Roelsgaard Jakobsen**

Denmark

**William James**

United Kingdom

**Marianne Jansson**

Sweden

**Anthony Jaworowski**

Australia

**Darlix Jean Luc**

France

**Rienk Jeeninga**

Netherlands

**Shibo Jiang**

China

**Dong-Yan Jin**

Hong Kong

**Gary Johanning**

United States of America

**Mina John**

Australia

**Marc Johnson**

United States of America

**Clare Jolly**

United Kingdom

**Sarah Joseph**

United States of America

**Georgette Kanmogne**

United States of America

**John Kappes**

United States of America

**Jonathan Karn**

United States of America

**Fatah Kashanchi**

United States of America

**Aris Katzourakis**

United Kingdom

**Ruian Ke**

United States of America

**Brandon Keele**

United States of America

**Stephen Kent**

Australia

**Julia Kenyon**

United Kingdom

**Arifa Khan**

United States of America

**Rosemary Kiernan**

France

**Frank Kirchhoff**

Germany

**Andrea Kirmaier**

United States of America

**P.J. Klasse**

United States of America

**Nichole Klatt**

United States of America

**Thomas Klimkait**

Switzerland

**Renate Koenig**

Germany

**Madhavi Kolli**

United States of America

**Jan Konvalinka**

Czech Republic

**Gerrit Koopman**

Netherlands

**Dusan Kordis**

Slovenia

**Yoshio Koyanagi**

Japan

**Christine Kozak**

United States of America

**Marit Kramski**

Australia

**Isaac Krauss**

United States of America

**Olaf Kutsch**

United States of America

**Mamuka Kvaratskhelia**

United States of America

**Jesse Kwiek**

United States of America

**Aksana Labokha**

United Kingdom

**Bernard Lafont**

United States of America

**Nadine Laguette**

France

**Nathaniel Landau**

United States of America

**Alan Landay**

United States of America

**Marie Larsson**

Sweden

**Stuart Le Grice**

United States of America

**Kelly Lee**

United States of America

**Edwin Leeansyah**

Sweden

**Andrew Leigh Brown**

United Kingdom

**Thomas Leitner**

United States of America

**Alice Len**

United Kingdom

**Jack Lenz**

United States of America

**Jeanette Leusen**

Netherlands

**Sharon Lewin**

Australia

**Chen Liang**

Canada

**Mathias Lichterfeld**

United States of America

**Jaisri Lingappa**

United States of America

**Maxine Linial**

United States of America

**Manuel Llano**

United States of America

**J. Stephen Lodmell**

United States of America

**Martin R. B. Loechelt**

Germany

**Jeremy Luban**

Switzerland

**Paolo Lusso**

United States of America

**Michael Malim**

United Kingdom

**François Mallet**

France

**Fabrizio Mammano**

France

**Nicolas Manel**

France

**Nicholas Maness**

United States of America

**Louis Mansky**

United States of America

**Youdong Mao**

United States of America

**Alessandro Marcello**

Italy

**David Margolis**

United States of America

**David Markovitz**

United States of America

**Juan Martin-Serrano**

United Kingdom

**Preston Marx**

United States of America

**Tetsuro Matano**

Japan

**Hiroshi Matsuo**

United States of America

**Masao Matsuoka**

Japan

**Patrick Matthias**

Switzerland

**Myra McClure**

United Kingdom

**Laura McCoy**

United States of America

**Aine McKnight**

United Kingdom

**Paul McLaren**

Canada

**Luis Menendez-Arias**

Spain

**Bo Meng**

United Kingdom

**Jean-Michel Mesnard**

France

**Thibault Mesplede**

Canada

**Gilles Mirambeau**

Spain

**Shogo Misumi**

Japan

**David Montefiori**

United States of America

**Ronald Montelaro**

United States of America

**Eugenio Montini**

Italy

**Christiane Moog**

France

**Penny Moore**

South Africa

**Arnaud Moris**

France

**Ian Morison**

New Zealand

**Lynn Morris**

South Africa

**Donald Mosier**

United States of America

**Walther Mothes**

United States of America

**Andrew J Mouland**

Canada

**Guillaume Mousseau**

United States of America

**Jan Münch**

Germany

**Carsten Münk**

Germany

**Tsutomu Murakami**

Japan

**Delphine Muriaux**

France

**Brian Murphy**

United States of America

**Venugopal Nair**

United Kingdom

**Richard Neher**

Germany

**Stuart Neil**

United Kingdom

**Monique Nijhuis**

Netherlands

**Kazuo Nishigaki**

Japan

**Philip Norris**

United States of America

**Mahdad Noursadeghi**

United Kingdom

**Vlad Novitsky**

United States of America

**David O'Connor**

United States of America

**Shelby O'Connor**

United States of America

**Shinya Oishi**

Japan

**Akira Ono**

United States of America

**Marcel Ooms**

United States of America

**Mario Ostrowski**

Canada

**Melanie Ott**

United States of America

**David Ott**

United States of America

**Dominique Ouellet**

Canada

**Mirko Paiardini**

United States of America

**Jean-Christophe Paillart**

France

**Marie Pancera**

United States of America

**Aridaman Pandit**

Netherlands

**Maria Papathanasopoulos**

South Africa

**Dimitrios Paraskevis**

Greece

**Leslie Parent**

United States of America

**Vinay Pathak**

United States of America

**William Paxton**

Netherlands

**Brendan Payne**

United Kingdom

**Finn Skou Pedersen**

Denmark

**Martine Peeters**

France

**Boris Matija Peterlin**

United States of America

**Karin Peterson**

United States of America

**Son Truong Pham**

Australia

**Vincent Piguet**

United Kingdom

**Cynthia Pise-Masison**

United States of America

**Mauro Pistello**

Italy

**Vicente Planelles**

United States of America

**Stefan Pöhlmann**

Germany

**Guido Poli**

Italy

**Filippos Porichis**

United States of America

**Owen Pornillos**

United States of America

**Kholoud Porter**

United Kingdom

**Pantelis Poumbourios**

Australia

**Christopher Power**

Canada

**Vinayaka Prasad**

United States of America

**David Price**

United States of America

**Waseem Qasim**

United Kingdom

**Peter Quashie**

Canada

**Nadia Rahm**

France

**Tariq Rana**

United States of America

**Charani Ranasinghe**

Australia

**Roland Regoes**

Switzerland

**Jan Rehwinkel**

United Kingdom

**Alan Rein**

United States of America

**Axel Rethwilm**

Germany

**Andrew Rice**

United States of America

**Laura Richert-Spuhler**

United States of America

**Espen Rimstad**

Norway

**Charles Rinaldo**

United States of America

**Harriet Robinson**

United States of America

**Michael Roche**

Australia

**Olivier Rohr**

France

**Nikolaus Romani**

Austria

**Susan Ross**

United States of America

**Edina Rosta**

United Kingdom

**Kenneth Roux**

United States of America

**Ioulia Rouzina**

United States of America

**Sarah Rowland-Jones**

United Kingdom

**Walter Royal**

United States of America

**Ruth Ruprecht**

United States of America

**Akihide Ryo**

Japan

**Ivan Sadowski**

Canada

**Asier Saez-Cirion**

France

**Manish Sagar**

United States of America

**Mineki Saito**

Japan

**Jun-ichi Sakuragi**

Japan

**Hiroaki Sakurai**

Japan

**Marco Salemi**

United States of America

**Rogier Sanders**

Netherlands

**Mario Santiago**

United States of America

**Nuno Santos**

Portugal

**Sarah Sasson**

Australia

**Quentin Sattentau**

United Kingdom

**Andrea Savarino**

Italy

**Bassel E Sawaya**

United States of America

**Torsten Schaller**

Germany

**Michael Schindler**

Germany

**Eric Schirmer**

United Kingdom

**Timothy Schlub**

Australia

**Jean-Claude Schmit**

Luxembourg

**Dominique Schols**

Belgium

**Olivier Schwartz**

France

**Robin Shattock**

United Kingdom

**Ann Sheehy**

United States of America

**Robert Siliciano**

United States of America

**Viviana Simon**

United States of America

**Richard David Sloan**

United Kingdom

**Nicolas Sluis-Cremer**

United States of America

**Ellen Sparger**

United States of America

**Paul Spearman**

United States of America

**Roberto Speck**

Switzerland

**Mario Stevenson**

United States of America

**Carol Stocking**

Germany

**Jonathan Stoye**

United Kingdom

**Klaus Strebel**

United States of America

**Hendrik Streeck**

United States of America

**Justine Sunshine**

United States of America

**Shinya Suzu**

Japan

**Valentina Svicher**

Italy

**Michael Swanson**

United States of America

**Yasuhiro Takeuchi**

United Kingdom

**Masafumi Takiguchi**

Japan

**Jun Jie Tan**

Malaysia

**Jamal Tazi**

France

**Philip Tedbury**

United States of America

**Amalio Telenti**

United States of America

**Emiliano Tesoro-Cruz**

Mexico

**Georgia Tomaras**

United States of America

**Sodsai Tovanabutra**

United States of America

**Simon Travers**

South Africa

**Alexandra Trkola**

Switzerland

**Athe Tsibris**

United States of America

**Mudit Tyagi**

United States of America

**Klaus Überla**

Germany

**Tonja van der Kuyl**

Netherlands

**Marit van Gils**

Netherlands

**Carine Van Lint**

Belgium

**Koen Van Rompay**

United States of America

**Anne-Mieke Vandamme**

Bermuda

**Bruno Verhasselt**

Belgium

**Theo Verrips**

Netherlands

**Beata G Vertessy**

Hungary

**Pornpun Vivithanaporn**

Thailand

**Volker M Vogt**

United States of America

**Thomas Vollbrecht**

United States of America

**Toshiki Watanabe**

Japan

**Irene Weber**

United States of America

**Leor Weinberger**

United States of America

**Carol Weiss**

United States of America

**Robin Weiss**

United Kingdom

**Sarah Welbourn**

United States of America

**Lucas Willems**

Belgium

**Sam Wilson**

United Kingdom

**Cheryl Winkler**

United States of America

**Li Wu**

United States of America

**Yuntao Wu**

United States of America

**Yoshihisa Yamano**

Jordan

**Ahmad Yatim**

France

**Venkat Yedavalli**

United States of America

**Xiao-Fang Yu**

United States of America

**Karina Yusim**

United States of America

**Jerome Zack**

United States of America

**Steven Zeichner**

United States of America

**Richard Y Zhao**

United States of America

**Yong-Hui Zheng**

United States of America

